# Probabilistic Forecasting and Information-Theoretic Analysis of Multivariate fMRI Dynamics

**DOI:** 10.3390/e28070738

**Published:** 2026-07-01

**Authors:** Arda Bayer, Zhiyao Zhang, Ahmet Emre Ipek, Rose Khavari, Behnaam Aazhang

**Affiliations:** 1 Department of Electrical & Computer Engineering, Rice University, Houston, TX 77005, USA; oz6@rice.edu (Z.Z.); aaz@rice.edu (B.A.); 2 Department of Electrical & Electronics Engineering, Özyeğin University, 34794 Istanbul, Türkiye; ipekahmetemre@gmail.com; 3 Department of Urology, Houston Methodist, Houston, TX 77030, USA; rkhavari@houstonmethodist.org

**Keywords:** functional magnetic resonance imaging, BOLD signal, probabilistic forecasting, information theory, entropy, directed information, brain dynamics, stochastic processes, transformer models, recurrent neural networks

## Abstract

Functional magnetic resonance imaging (fMRI) signals exhibit complex temporal structure arising from multivariate neural dynamics, physiological variability, and measurement uncertainty. In this work, we formulate region-of-interest-level fMRI analysis as a probabilistic multi-step forecasting problem and investigate the predictability of blood-oxygen-level-dependent (BOLD) activity from an information-theoretic perspective. Using the Natural Scenes Dataset, we model multiregional BOLD activity as a stochastic process with finite memory and train multiple forecasting architectures, including linear regression, exponential smoothing, recurrent neural networks, and transformer-based models, to predict future BOLD samples from preceding temporal observations. Forecasting performance is analyzed together with entropy-based quantities, including marginal entropy, conditional entropy, and normalized predictive information measures estimated directly from model-derived predictive distributions without imposing restrictive Gaussian assumptions on the underlying BOLD dynamics. The transformer model achieved significant improvement over a naive persistence baseline (p=0.001) while yielding a high predictive information fraction (η=75.49%). Post hoc directed information analysis revealed that short-horizon prediction was dominated primarily by autoregressive, within-ROI, temporal structure. Overall, the proposed framework demonstrates how probabilistic forecasting and information-theoretic analysis can be integrated to characterize the predictability, uncertainty structure, and directional organization of large-scale fMRI dynamics and may support future downstream neuroengineering and neural-state inference applications.

## 1. Introduction

Forecasting is fundamentally linked to the amount of information available about a dynamical system. A system that can be predicted accurately over future time horizons necessarily contains structured temporal dependencies that reduce uncertainty about future states. From an information-theoretic perspective, forecastability therefore provides an empirical measure of the complexity, organization, and predictability of the underlying process. Beyond characterization alone, accurate forecasting is also closely related to downstream tasks such as monitoring, control, and decision-making, where future system behavior must be estimated from incomplete observations.

In neuroscience, the ability to model and predict brain activity has become increasingly important with the rapid growth of brain-computer interfaces, neural decoding systems, and data-driven neurotechnology [1]. Recent advances in machine learning have demonstrated that complex cognitive and perceptual information can be inferred from neuroimaging signals, particularly functional magnetic resonance imaging (fMRI). For example, multiple studies have shown that visual stimuli [2,3], semantic representations, and even continuous language content can be reconstructed from fMRI recordings using large-scale predictive models and deep learning frameworks [4]. These include image reconstruction approaches based on latent diffusion and self-supervised representations, as well as semantic decoding systems capable of recovering continuous language representations from non-invasive brain recordings [3,5]. Collectively, these works demonstrate that fMRI blood-oxygen-level-dependent (BOLD) activity contains substantial latent structure that can support complex predictive and decoding tasks.

The existence of such latent structure naturally raises a complementary question: how predictable are the underlying BOLD dynamics themselves? While most neuroimaging prediction studies have focused on application-driven objectives such as reconstruction or decoding accuracy [2,5], the forecasting problem can also be viewed from an information-theoretic perspective. In this setting, forecasting performance serves not only as a measure of predictive accuracy but also as a probe of the statistical organization of the BOLD signal and the extent to which future neural activity is constrained by prior temporal observations.

Related questions have previously been investigated through entropy-based analyses of fMRI activity. Prior studies have used entropy-derived measures to characterize the complexity and irregularity of BOLD signals [6,7,8]. While these approaches provide valuable descriptions of signal complexity, they do not directly quantify how much uncertainty about future neural activity can be reduced through knowledge of the past. Forecastability addresses this complementary question by measuring the predictive information shared between past and future observations. Accordingly, the present work focuses on quantifying the fraction of future BOLD uncertainty that can be explained from preceding temporal observations through a normalized predictive information measure.

In this work, we formulate multiregional fMRI forecasting as a probabilistic time-series prediction problem grounded in information theory. We model the BOLD signal as a multivariate stochastic process with finite temporal memory. Specifically, the process is represented using a memory parameter *M* and a prediction horizon *H*, where the task is to forecast the next *H* future samples using the preceding *M* observations across a set of predefined regions of interest (ROIs). Under this formulation, forecasting quality becomes directly related to the reduction in uncertainty achievable from past observations.

Using this framework, we investigate both the predictability and the information structure of ROI-level BOLD dynamics obtained from the Natural Scenes Dataset (NSD), a large-scale 7T fMRI dataset acquired during continuous natural scene viewing. We evaluate multiple forecasting architectures spanning classical statistical methods and modern machine learning approaches, including linear regression, exponential smoothing, recurrent neural networks, and transformer-based sequence models. Rather than focusing exclusively on predictive accuracy, we analyze forecasting performance through entropy-based quantities derived from the learned probabilistic models, including marginal entropy, conditional entropy, and normalized predictive information measures. In this setting, the forecasting models can also be interpreted as data-driven forward models of short-timescale brain dynamics, providing compact probabilistic representations of how future neural activity evolves from preceding observations. Such forward-model formulations are broadly relevant to downstream neuroengineering and neural-state inference problems, where future brain activity must be estimated from incomplete or noisy observations.

In addition to evaluating forecasting performance, we further investigated how predictive information was distributed across cortical regions using directed information (DI). Whereas forecasting accuracy alone quantifies how well future BOLD activity can be predicted, DI provides a complementary view into which ROI histories contribute most strongly to those predictions and how predictive structure propagates across the network. In this sense, the forecasting models can be interpreted not only as predictive tools but also as data-driven probes of directional statistical organization in multivariate brain dynamics. Directed information extends mutual information to temporally ordered stochastic processes and quantifies the extent to which the past activity of one process contributes to predicting the future activity of another process beyond self-history effects [9]. Related information-theoretic frameworks for dependence and information flow analysis have been studied extensively in information theory and network inference, including work associated with directed information and causal dependence measures in stochastic dynamical systems [10,11,12]. In the present study, DI was estimated post hoc from the trained probabilistic forecasting models, enabling directional analysis of predictive dependencies directly from the learned forecasting distributions.

The forecasting perspective adopted here is also motivated by broader developments in predictive modeling and sequential inference. In large-scale forecasting benchmarks such as the M5 forecasting competition, forecasting accuracy has been shown to depend strongly on the interaction between model structure, uncertainty estimation, and temporal dependencies in the underlying data [13]. Similar principles arise in neural systems, where the ability to predict future states may provide insight into the complexity and organization of brain dynamics [14,15]. By combining probabilistic forecasting with entropy and directed information analysis, the present work aims to provide a unified framework for studying the predictability, uncertainty structure, and directional organization of multivariate fMRI activity, while also motivating forecasting-based forward models for downstream neuroengineering and neural-state inference applications.

## 2. Materials and Methods

This section presents the dataset, preprocessing procedures, forecasting models, and information-theoretic analysis framework used in this study. Forecastability and directed dependencies were analyzed from an information-theoretic perspective, building on broader frameworks for inference and dependence analysis in complex stochastic systems [9,16].

### 2.1. Dataset and Region-of-Interest Definition

Functional magnetic resonance imaging (fMRI) data were obtained from the NSD [17], a large-scale 7T fMRI dataset acquired during continuous visual stimulation with natural scene images. NSD contains one of the largest amounts of single-subject fMRI data currently publicly available, enabling high-resolution analysis of long-timescale cortical dynamics. In this work, we used the publicly released preprocessed 1.8 mm isotropic BOLD time series provided as part of the NSD preprocessing pipeline. Data from eight subjects were included. The NSD dataset is organized hierarchically into imaging sessions and individual functional runs, where each session contains multiple continuous BOLD acquisitions corresponding to separate stimulus presentation blocks. The present study focused on the visual cortex due to its strong stimulus-driven responses, well-characterized hierarchical organization, and suitability for studying multiscale predictive structure in brain dynamics.

A total of 23 visual cortical ROIs were defined in Montreal Neurological Institute (MNI) space using spherical masks centered on canonical visual-system coordinates. ROIs were defined as spherical regions centered on approximate MNI coordinates corresponding to established visual cortical areas identified in probabilistic retinotopic atlases and prior functional localization studies [18,19,20,21]. The primary visual cortex V1 was represented by a single midline ROI, whereas all remaining regions were represented bilaterally, resulting in 23 ROIs in total. Early visual cortex regions, V1, V2, and V3, were assigned a radius of 5 mm, while higher-order visual areas used a radius of 6 mm as described in Table 1.

The ROI set spans multiple stages of the human visual system, including early retinotopic cortex (V1–V3), intermediate dorsal and ventral retinotopic areas (V3A, V3B, and hV4), lateral occipital retinotopic maps (LO1 and LO2), ventral occipital maps (VO1 and VO2), and category-selective ventral temporal regions, including the parahippocampal place area (PPA) and fusiform face area (FFA). The ROI coordinates were selected based on established visual neuroanatomical landmarks and prior retinotopic mapping literature [18,19,20,21].

To obtain subject-specific ROI time series, each ROI atlas defined in MNI space was nonlinearly warped into each subject’s anatomical and functional space using ANTs-based registration [22]. Specifically, MNI-to-subject anatomical registration used symmetric normalization, followed by affine alignment between the subject anatomical and functional scan coordinate spaces. Mean BOLD activity within each ROI was then extracted for every timepoint of every run.

### 2.2. Forecasting Problem Formulation

Let $X_{t}\in R^{N}$ denote the multivariate BOLD signal across *N* ROIs at discrete time index *t*. Throughout this work, uppercase symbols such as $X_{t}$ denote random variables or stochastic processes, whereas lowercase symbols such as $x_{t}$ denote observed realizations of those variables. In addition, *Y* is used to denote the target random variable corresponding to future values of the underlying BOLD process $X_{t}$. Given a memory length $M\in Z^{+}$, a positive integer, and forecasting horizon $H\in Z^{+}$, the objective is to learn a forecasting function $f:R^{M\times N}\to R^{H\times N}$ that maps a window of past BOLD observations $\left(X_{t-M+1},\ldots ,X_{t}\right)$ to a prediction of future activity $\left(X_{t+1},\ldots ,X_{t+H}\right)$. The forecasting problem was therefore formulated as supervised multivariate sequence prediction. Forecasting performance was interpreted as an empirical probe of the predictability and information structure of fMRI dynamics for the selected ROI.

Model performance was evaluated using multiple complementary metrics. Root mean squared error (RMSE) was used as the primary forecasting accuracy metric. RMSE is widely used in time-series forecasting studies as a relative measure for comparing competing predictive models rather than as an absolute measure of physical signal fidelity. Accordingly, the primary interpretation of RMSE in the present work is through comparisons between forecasting approaches and against naive persistence baselines. We additionally evaluated the Root Mean Squared Scaled Error (RMSSE) metric introduced in the M5 forecasting competition [13], where prediction error is normalized relative to a naive one-step forecasting baseline.

The normalized predictive information fraction was defined as

(1) $$\eta =1-\frac{H(Y|X)}{H(Y)}=\frac{I(X;Y)}{H(Y)},$$

which corresponds to an asymmetric normalized mutual information measure related to the uncertainty coefficient in information theory [23,24]. Here, $Y=(X_{t+1},\ldots ,X_{t+H})$ is the random variable corresponding to the future BOLD signal over the forecasting horizon *H*, and $X=(X_{t-M+1},\ldots ,X_{t})$ is the random variable associated with the past observed signal window. For notational simplicity, entropy and mutual information quantities are written without explicit time dependence, implicitly assuming time-invariant joint statistics over the sampled forecasting windows. Here, H(·) denotes Shannon entropy and H(·|·) the conditional Shannon entropy, and I(X;Y) denotes the mutual information between past and future BOLD activity [23]. By definition, η∈[0,1], where larger values indicate greater predictability of future signal dynamics from past observations. Equivalently, η can be interpreted as the relative reduction in expected coding length of the target signal *Y* when the past signal *X* is known compared to when it is unknown.

The marginal entropy and conditional entropy were estimated as

(2) $$H\left(Y\right)\approx E\left[-\log p_{\theta }\left(Y\right)\right],\;H\left(Y|X\right)\approx E\left[-\log p_{\theta }\left(Y|X\right)\right],$$

where $p_{\theta }\left(Y\right)$ and $p_{\theta }\left(Y|X\right)$ denote the discretized empirical marginal and discretized model-predicted conditional probability distributions, respectively. The marginal distribution $p_{\theta }\left(Y\right)$ was estimated using a histogram-based discrete density estimator with 100 bins fit on the training portion of the dataset. To estimate the conditional distribution $p_{\theta }\left(Y|X\right)$, predictive residuals obtained from the trained forecasting models were discretized using the same histogram binning procedure. Let $\hat{Y}=f_{\theta }\left(X\right)$ denote the model prediction and define the residual variable $R=Y-\hat{Y}$. Empirical residual histograms were estimated independently for each ROI and forecasting horizon from calibration residuals obtained on held-out validation data. The conditional predictive distribution was then approximated as

(3) $$p_{\theta }\left(Y|X\right)\approx p_{R}\left(Y-f_{\theta }\left(X\right)\right),$$

where $p_{R}\left(\cdot \right)$ denotes the discretized empirical residual distribution. This histogram-based construction enabled nonparametric approximation of conditional predictive likelihoods without imposing Gaussian assumptions on the forecasting residuals. Using the model-derived conditional distributions enabled estimation of η directly from the predictive structure learned by the forecasting models, thereby characterizing the information captured by the fitted dynamical representations.

### 2.3. Data Parsing and Sliding-Window Construction

Each fMRI run was independently normalized using run-level z-score normalization applied separately to each ROI time series:

(4) $${\tilde{x}}_{t}^{i}=\frac{x_{t}^{i}-\mu_{i}}{\sigma_{i}+\epsilon },$$

where $x_{t}^{i}$ is the BOLD signal for ROI *i* at discrete time *t*, $\mu_{i}$ and $\sigma_{i}$ denote the within-run [17] mean and $\sigma_{i}$ standard deviation of ROI *i*. This normalization avoids distribution leakage across subjects during leave-one-subject-out evaluation.

The normalized time series were transformed into supervised learning samples using an overlapping sliding-window procedure. For each run, input windows of length *M* and target windows of length *H* were extracted using an overlapping sliding-window procedure with one-sample increments. Specifically,

(5) $$x_{t}=\left[{\tilde{x}}_{t},\ldots ,{\tilde{x}}_{t+M-1}\right]\;\mathrm{and}\;y_{t}=\left[{\tilde{x}}_{t+M},\ldots ,{\tilde{x}}_{t+M+H-1}\right]$$

where ${\tilde{x}}_{t}$ is the normalized BOLD signal for *N* ROIs. Aggregating the windowed samples extracted across all NSD runs and sessions yielded the supervised forecasting dataset consisting of the input tensor x and target tensor y.

This procedure converts each continuous multivariate BOLD sequence into a large collection of partially overlapping forecasting examples. Subject identities were preserved during splitting to prevent information leakage between training and testing partitions.

### 2.4. Forecasting Models

Four forecasting approaches spanning classical statistical models and modern deep learning architectures were evaluated.

#### 2.4.1. Linear Regression

A ridge-regularized multivariate linear regression model [25] was used as one of the primary forecasting baselines. For each forecasting sample, the input window of shape M×N was flattened into a single feature vector of dimension MN, allowing the model to jointly learn temporal and cross-ROI relationships from the past BOLD activity. The regression model then learned a direct mapping from the flattened input history to the future forecasting target over the prediction horizon.

The linear forecasting model was parameterized by a weight tensor $W\in R^{MN\times HN}$ defining a linear mapping $f_{W}$ from the input history tensor $x_{t}\in R^{M\times N}$ to the future target tensor $y_{t}\in R^{H\times N}$. Given a collection of *n* supervised forecasting samples ${\left\{\left(x_{t},y_{t}\right)\right\}}_{t=1}^{n}$, ridge regularization was used to estimate the forecasting weights:

(6) $$\hat{W}=\underset{W}{arg min}\sum_{t=1}^{n}\left|\right|y_{t}-f_{W}\left(x_{t}\right){\left|\right|}_{F}^{2}+{\alpha ||W||}_{F}^{2},$$

where $\left|\right|\cdot {\left|\right|}_{F}$ denotes the Frobenius norm and α=1.0 controls the regularization strength. Ridge regularization was chosen to stabilize estimation in the high-dimensional forecasting setting, particularly since the number of temporal forecasting features grows proportionally with both the memory length *M* and the number of ROIs *N*.

Although substantially simpler than deep neural forecasting architectures, the linear regression model can still capture a considerable portion of the temporal structure present in the BOLD signals. Comparisons between the forecasting performance of the linear model and higher-capacity nonlinear models provide insight into the underlying signal complexity and noise characteristics of the data. In particular, similar performance between linear and nonlinear models may indicate that the observed dynamics are dominated by approximately linear dependencies or by variability arising from unobserved sources that cannot be effectively modeled even by overparameterized nonlinear architectures.

#### 2.4.2. Exponential Smoothing

Exponential smoothing is a classical time-series forecasting approach that recursively estimates future observations using weighted averages of past measurements, assigning progressively greater weight to more recent samples. Compared to the neural network architectures evaluated in this work, exponential smoothing provides a substantially simpler statistical forecasting baseline primarily intended to capture short-timescale autoregressive structure in ROI activity.

The exponential smoothing model, together with appropriate trend and seasonality extensions, served as a strong baseline in the M5 forecasting competition [13], where the relatively simple statistical method was shown to outperform many more elaborate machine learning approaches [13]. Since the fMRI BOLD signals analyzed in this study did not exhibit a consistent global trend or periodic seasonal structure, no explicit trend or seasonal components were included in the model. Consequently, the implemented formulation corresponds to the *Simple Exponential Smoothing* (SES) model [26], in which future predictions are generated solely from exponentially weighted averages of past observations.

The simple exponential smoothing forecastor can be described as [26]

(7) $$\begin{array}{cc} {\hat{x}}_{t} & =\ell_{t-1} \end{array}$$

(8) $$\begin{array}{cc} \ell_{t} & =\alpha x_{t}+\left(1-\alpha \right)\ell_{t-1}, \end{array}$$

where $x_{t}$ is the observed signal value corresponding to the random variable $X_{t}$, $\ell_{t}$ is the latent variable, α∈[0,1] is the smoothing parameter and ${\hat{x}}_{t}$ is the model prediction. Once a prediction is obtained for time instance *t*, it is recursively used as the observed signal to infer the remaining future values ${\hat{x}}_{t+h}$ over the horizon *H* for h=1,…,H−1. To ensure comparability with the other models, the latent variable is initialized such that $\ell_{t-m}=0$ for m>M, while $\ell_{0}$ was set to the mean BOLD signal value computed from the training data.

Unlike the other forecasting models evaluated in this work, exponential smoothing does not naturally support multivariate inputs in which multiple ROI time series are jointly used to predict future activity. Consequently, a separate exponential smoothing model was fit independently for each ROI using only its own preceding temporal observations. This yields a comparatively low-complexity forecasting framework in which each ROI model is parameterized primarily by a single smoothing coefficient, α, controlling the relative weighting of recent versus past observations.

#### 2.4.3. Long Short-Term Memory Network

To capture nonlinear temporal dependencies, we implemented a multi-layer Long Short-Term Memory (LSTM) network [27]. The model receives an input tensor of shape (M,N), where *M* denotes the temporal memory window and *N* the number of ROIs. The architecture consisted of three recurrent LSTM layers with a hidden-state dimension of 512 units and an inter-layer dropout probability of 0.5 to reduce overfitting. The recurrent layers were configured using batch-first ordering, enabling direct processing of ROI time-series windows.

The hidden state of the final recurrent layer at the last observed time point was passed to a fully connected projection layer that maps the latent representation into the full forecasting horizon. Specifically, the final linear layer outputs a vector of dimension H×N, which is reshaped into a prediction tensor of shape (H,N) corresponding to simultaneous multi-step forecasts across all ROIs. The training protocol details, including the training loss and the optimizer pick, are given in Section 2.5.

#### 2.4.4. Transformer

We further evaluated a transformer-based sequence model employing self-attention mechanisms for long-range temporal dependency modeling [28]. The transformer architecture operated on input windows of shape (M,N) and first projected each ROI vector into a latent embedding space of dimension $d_{\mathrm{model}}=64$ using a learned linear projection layer. Learned positional embeddings were added to preserve temporal ordering information within the sequence.

The embedded sequence was processed using an encoder-only transformer composed of two stacked transformer encoder layers with four attention heads per layer and dropout probability 0.1. The encoder outputs contextualized latent representations for all temporal positions. The latent representation corresponding to the final input time point was then passed through a linear output layer, producing a vector of dimension H×N, which was reshaped into the multi-step prediction tensor of shape (H,N). This formulation enables the model to capture distributed temporal interactions between ROIs through self-attention on the concatenated input space, $R^{M\times N}$, while directly generating the full forecasting horizon in a single forward pass. The training protocol for this neural network is also provided in Section 2.5.

### 2.5. Training and Evaluation Protocol

Model evaluation followed a two-stage subject-level generalization protocol. First, two subjects were reserved as a fully held-out test set and excluded entirely from model selection and cross-validation procedures. The remaining six subjects were then used for leave-one-subject-out cross-validation (LOSO-CV), where in each fold one subject served as the validation subject while the remaining subjects were used for training. This LOSO framework enabled assessment of cross-subject generalization while reducing the risk of subject-specific overfitting. For neural forecasting models, the best-performing model weights from the LOSO folds, determined using validation performance and early stopping, were subsequently evaluated on the previously unseen held-out test subjects to obtain the final test results.

For deep learning models, training employed the AdamW optimizer [29] with an initial learning rate of $5\times 10^{-4}$ and weight decay of $10^{-5}$. Adaptive learning-rate scheduling was performed with multiplicative decay factor 0.5 [30], while early stopping based on validation loss was used to reduce overfitting. The training objective combined a Huber reconstruction loss for the predicted BOLD time series [31] with an additional temporal-difference regularization term that penalizes discrepancies in first-order temporal dynamics between predicted and observed signals:

(9) $$\begin{array}{cc} L(x_{t},{\hat{x}}_{t};\delta ) & =L_{Huber}\left(x_{t},{\hat{x}}_{t};\delta \right)+\alpha L_{\Delta }\left(x_{t},{\hat{x}}_{t};\delta \right) \end{array}$$

(10) $$\begin{array}{cc} L_{\Delta }\left(x_{t},{\hat{x}}_{t};\delta \right) & =L_{Huber}\left(x_{t}-x_{t-1},{\hat{x}}_{t}-{\hat{x}}_{t-1};\delta \right), \end{array}$$

where α=0.3 controls the contribution of the temporal-difference regularization term, δ=0.5 denotes the Huber threshold parameter, and $L_{\Delta }$ penalizes discrepancies between predicted and observed temporal differences across adjacent forecast steps. For completeness, the Huber reconstruction loss was defined as

(11) $$\begin{array}{cc} L_{Huber}\left(x_{t},{\hat{x}}_{t};\delta \right) & =\frac{1}{H}\sum_{h=0}^{H-1}\phi_{\delta }\left(x_{t+h}-{\hat{x}}_{t+h}\right) \end{array}$$

(12) $$\begin{array}{cc} \phi_{\delta }\left(z\right) & =\left\{\begin{array}{c} \frac{1}{2}z^{2},\;\left|z\right|\leq \delta \\ \delta (|z|-\frac{1}{2}\delta ),\;|z|>\delta \end{array}\right.. \end{array}$$

where *H* denotes the forecasting horizon. We systematically varied the memory parameter *M* and forecasting horizon *H* to characterize the temporal predictability structure of the fMRI signals. Model selection was based on cross-validated forecasting performance across LOSO folds.

Forecasting results were additionally compared against a naive persistence baseline that repeats the last observed frame over the prediction horizon. Statistical significance was assessed using a permutation test applied to the transformer forecasting model, which was selected as a representative forecasting architecture for the subsequent entropy and directed information analyses. This choice illustrates that the proposed information-theoretic framework remains applicable and interpretable even when coupled with a comparatively high-capacity nonlinear forecasting model. The purpose of the permutation test was to verify that the observed forecasting performance exceeded that expected under random target-label associations. The test statistic was the aggregate RMSE computed across all test samples, ROIs, and forecast horizons, yielding a single scalar measure of forecasting accuracy. A null distribution was generated using 1000 permutations in which the training targets were randomly permuted, and the forecasting model retrained. The reported *p*-value therefore corresponds to a single global hypothesis test comparing overall forecasting performance against the naive persistence baseline. Because separate hypothesis tests were not performed across individual ROIs, forecast horizons, or model classes, no multiple-comparison correction was applied.

### 2.6. Directed Information Post Analysis

To investigate directed dependencies between ROIs, we performed a post hoc DI analysis using the trained forecasting models. DI provides a directional information-theoretic measure quantifying the extent to which the past activity of one source random variable contributes to predicting the future activity of a target random variable [9]. In contrast to forecasting accuracy alone, which quantifies overall predictability of future BOLD activity, DI provides a complementary view into which ROI histories contribute most strongly to those predictions and how predictive structure propagates across the network. Related information-theoretic frameworks for analyzing dependence and information flow in complex systems have also been studied extensively within the information theory literature [16].

For each source ROI signal $X^{i}$ and target ROI signal $X^{j}$, we estimated pairwise marginal directed information by comparing predictive likelihoods obtained from the full model against a reduced-input model in which the source ROI history was removed. Specifically, the estimator takes the form [9,10,32]

(13) $$I\left(X^{i}\to X^{j}\right)=E\left[\log p\left(X_{t+1}^{j}|H_{t}\right)-\log p\left(X_{t+1}^{j}|H_{t}^{-i}\right)\right],$$

where p(A|B) denotes the conditional probability distribution of a random variable *A* given another random variable *B*, $H_{t}$ denotes the complete multivariate history, and $H_{t}^{-i}$ denotes the history with ROI *i* removed. As in the entropy analysis, the expectation was estimated over pooled forecasting samples under an implicit assumption of time-invariant joint statistics across the sampled windows. The probabilistic predictive distributions were constructed post hoc using empirical residual histogram models. Let ${\hat{X}}_{t+1}^{j}=f_{\theta }\left(H_{t}\right)$ denote the model prediction for ROI *j* at forecasting horizon h=1, and define the residual variable

(14) $$R_{t}^{j}=X_{t+1}^{j}-{\hat{X}}_{t+1}^{j}.$$

**Figure 1:**
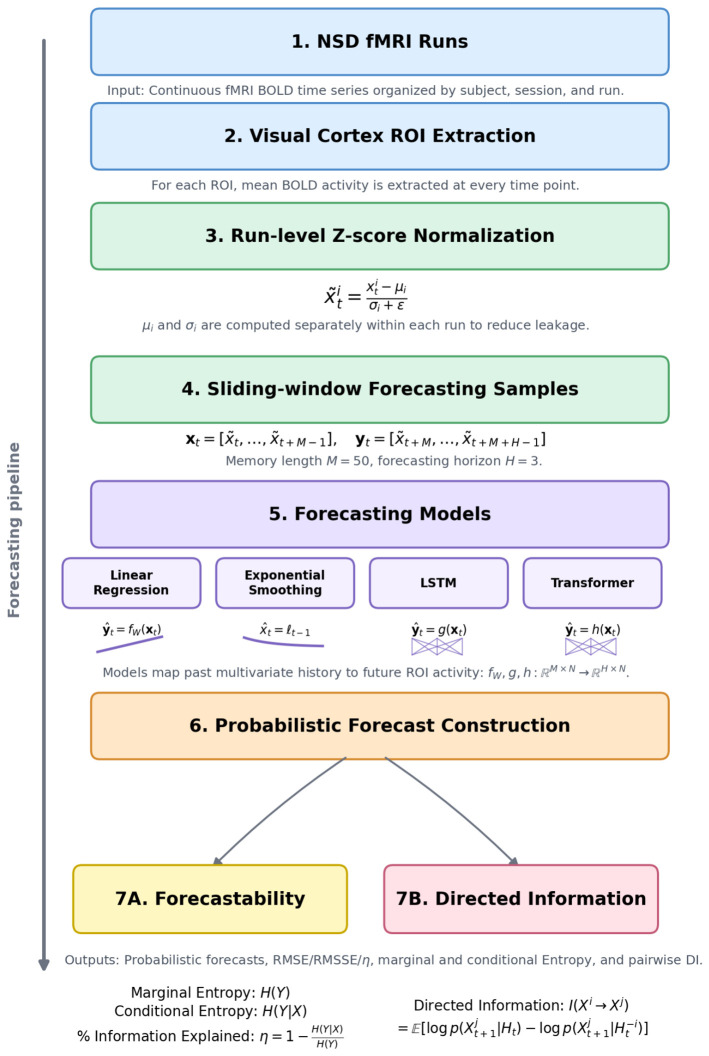
Overview of the proposed multivariate fMRI forecasting framework. Preprocessed BOLD time series extracted from predefined regions of interest (ROIs) were converted into sliding temporal windows and used to train forecasting models to predict future ROI activity from past observations. Forecasting performance and predictive distributions were subsequently used to compute error-based and information-theoretic measures, including conditional entropy and directed information.

For each ROI and forecasting horizon, empirical residual distributions were estimated from calibration residuals using histogram-based discrete density estimation. The conditional predictive distribution was then approximated as

(15) $$p\left(X_{t+1}^{j}|H_{t}\right)\approx p_{R}\left(X_{t+1}^{j}-f_{\theta }\left(H_{t}\right)\right),$$

where $p_{R}\left(\cdot \right)$ denotes the empirical residual probability mass function estimated from the calibration data. This construction enabled nonparametric sample-based approximation of conditional predictive likelihoods without imposing Gaussian assumptions on the forecasting residuals, thereby allowing directed information estimation from arbitrary forecasting architectures. The whole forecasting pipeline is summarized in Figure 1.

## 3. Results

The multi-model forecasting framework was evaluated for multi-step prediction of ROI-level BOLD dynamics. Forecasting models were trained using leave-one-subject-out cross-validation with windowed BOLD sequences as input and future ROI activity as the prediction target.

**Figure 2:**
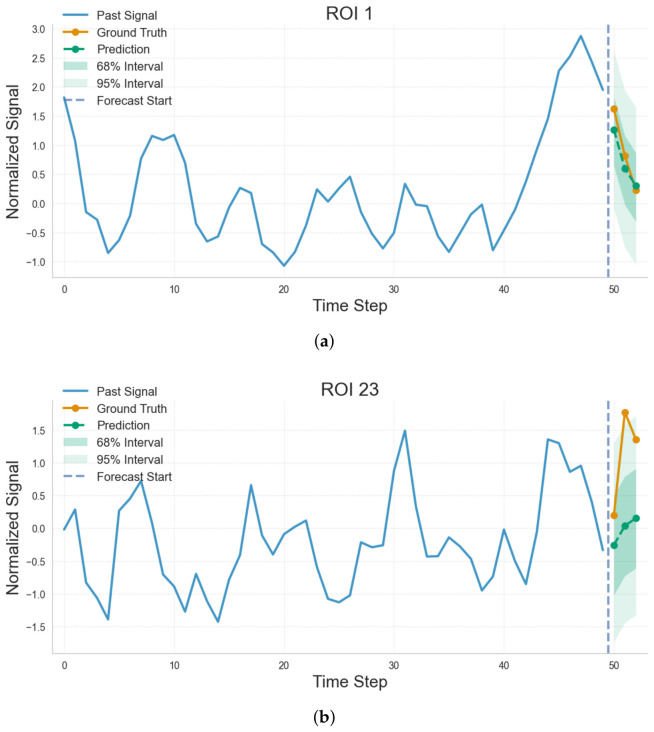
Illustration of probabilistic forecasting performance for two representative ROIs. The historical ROI activity preceding the forecast horizon is shown together with the ground-truth future trajectory, model prediction, and uncertainty intervals estimated from forecasting residuals. (**a**) Example of a well-predicted ROI trajectory, where the model accurately captures the temporal evolution of the BOLD signal within the forecast horizon. (**b**) Example of a poorly predicted ROI trajectory, illustrating increased forecasting error and uncertainty. These examples highlight the heterogeneous forecastability of fMRI dynamics across ROIs and temporal instances.

Figure 2 illustrates example three-step probabilistic forecasts generated by the transformer model for two representative ROIs. One example demonstrates relatively close agreement between the predicted and observed BOLD trajectories over the forecast horizon, whereas the second example highlights a case with larger prediction error. In both cases, the model provides a probabilistic estimate of future activity rather than a deterministic reconstruction of the signal. These examples emphasize that the forecasting framework captures statistical structure in the ROI dynamics while still exhibiting substantial uncertainty and imperfect predictive accuracy, consistent with the noisy and stochastic nature of fMRI BOLD measurements.

Forecasting performance across the evaluated architectures is summarized in Table 2, including both cross-validation results and performance on the hold-out test subjects. The forecasting performances were comparable, and the linear model achieved the highest overall predictive performance among the validated approaches.

**Figure 3:**
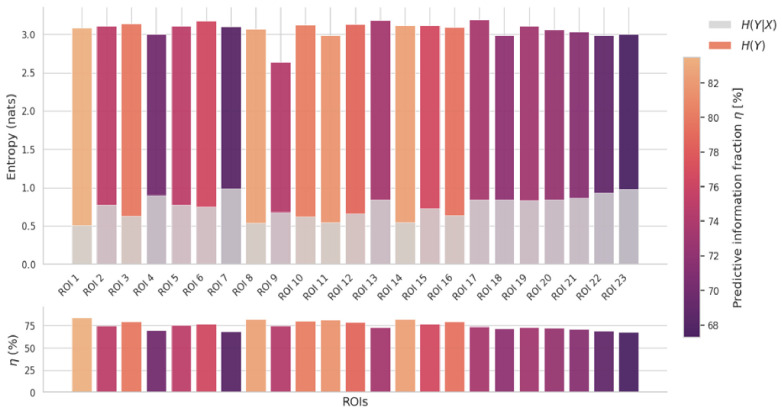
Forecastable information across ROIs for the transformer model. **Top Figure**: Barplot shows the information content of the target BOLD signal measured by empirical Shannon Entropy H(Y). The conditional entropy H(Y|X) of the target BOLD signal *Y* given past signal *X* is given in gray. The difference H(Y)−H(Y|X) is the forecastable information and color codes the percent of forecastable information per ROI. **Bottom Figure**: Percent forecastable information η=1−H(Y|X)/H(Y) per ROI.

To quantify the information content captured by the forecasting framework, we computed entropy-based measures from the probabilistic forecasts. The transformer model achieved a mean marginal entropy of H(Y)=3.07 nats and a mean conditional entropy of H(Y|X)=0.75 nats across ROIs, yielding an average predictive information gain of H(Y)−H(Y|X)=2.32 nats. The corresponding normalized predictive information fraction was η=75.49% for the multivariate problem. Per-ROI entropy decomposition values are shown in Figure 3, where marginal entropy, conditional entropy, and normalized predictive information are visualized jointly for each ROI.

A permutation-based significance test using 1000 target-shuffled surrogate models demonstrated that the forecasting performance was highly unlikely to arise from arbitrary associations between past and future observations (p≤0.001). Similar results were obtained for both transformer and linear forecasting models.

To further investigate the origin of the observed predictive information, we performed a phase-randomization control analysis. For each ROI, Fourier phases were randomized while preserving the original amplitude spectrum, thereby maintaining the signal variance and power spectral density while destroying temporal phase relationships. Repeating the forecasting and entropy analysis on the resulting surrogate data reduced the normalized predictive information fraction from η=75.49% to η=5.18%. This substantial reduction indicates that the predictive information captured by the forecasting models depends primarily on temporally organized structure in the BOLD signal rather than on second-order spectral statistics alone.

### Post Hoc Directed Information Analysis

Following model training, a post hoc DI analysis was performed using the probabilistic forecasting outputs. The DI computation compared predictive likelihoods obtained using the full ROI history against likelihoods obtained after selectively removing the history of individual source ROIs. This yielded a directed information matrix quantifying the contribution of each source ROI to predicting each target ROI.

**Figure 4:**
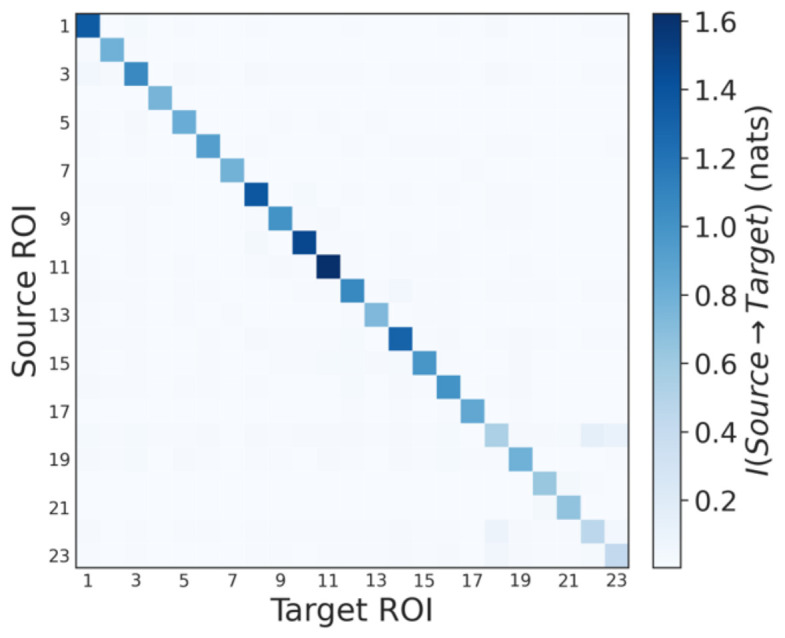
Directed information (DI) matrix estimated from the transformer model (M=50, H=3) across the selected ROIs. Larger values indicate stronger directional predictive contributions from the source ROI (rows) to the target ROI (columns). The predominantly diagonal structure suggests that short-horizon BOLD forecasting is dominated primarily by autoregressive within-ROI temporal dependencies, whereas cross-ROI directed interactions remain comparatively weaker.

The resulting DI matrix for the transformer model is shown in Figure 4. For the selected memory length M=50 and forecast horizon H=3, the matrix exhibited a predominantly diagonal structure, indicating that the strongest predictive contributions arose from within-ROI temporal history rather than cross-ROI interactions. This finding was consistent among the forecasting models tested in this work.

To assess the sensitivity of these findings to the selected forecasting timescale, additional DI analyses were performed using memory lengths *M* = 25, 50, and 75 and forecasting horizons *H* = 1, 3, and 5. Across the tested parameter settings, the resulting DI matrices remained predominantly diagonal, with only modest variations in the absolute DI values. No substantial increase in off-diagonal cross-ROI directed interactions was observed. These results suggest that the dominance of within-ROI temporal dependencies is robust to moderate changes in the forecasting memory and prediction horizon.

## 4. Discussion

The present study demonstrates that multivariate fMRI BOLD dynamics contain substantial short-horizon predictive structure that can be captured using probabilistic forecasting models. Across all evaluated approaches, forecasting performance significantly exceeded that of a naive persistence baseline (p=0.001), indicating that the models learned nontrivial temporal dependencies beyond simple signal persistence.

Entropy decomposition further showed that a substantial fraction of uncertainty in future BOLD activity could be reduced using the preceding ROI history. The relatively low conditional entropy compared to the marginal entropy indicates that short-timescale fMRI dynamics are not purely stochastic, but instead exhibit measurable temporal organization. At the same time, the persistence of nonzero conditional entropy suggests that a considerable component of the signal remains unpredictable, potentially reflecting measurement noise, physiological variability, latent neural states, or unobserved network processes not represented within the selected ROI set.

This interpretation is further supported by the phase-randomization control analysis. Randomizing Fourier phases while preserving the original power spectrum reduced the normalized predictive information fraction from 75.49% to 5.18%. Since phase randomization preserves signal variance and second-order spectral characteristics while eliminating temporal phase structure, this result suggests that the predictive information identified by the forecasting models arises primarily from temporally organized dependencies in the observed BOLD process rather than from spectral properties alone. At the same time, because the analysis was performed on the measured BOLD signal, the identified predictive structure should not be interpreted as uniquely neural in origin. Instead, it reflects the aggregate temporal organization of the recorded process, which may include neural, hemodynamic, physiological, and measurement-related contributions.

The ROI-level entropy analysis additionally revealed a heterogeneous predictive structure across the visual hierarchy. Marginal entropy values remained relatively consistent across ROIs, suggesting broadly similar overall signal variability throughout the visual cortex. In contrast, conditional entropy and predictive information fractions varied substantially between regions. Early and intermediate visual areas, including V1, V3A, V3B, and LO2, exhibited some of the highest predictability values, indicating strong local temporal continuity and relatively stable short-timescale dynamics. Conversely, higher-order ventral temporal regions, particularly the fusiform face area (FFA) and parahippocampal place area (PPA), demonstrated comparatively lower predictability and higher conditional entropy, suggesting increased stochastic variability or more complex latent dynamics. These findings are consistent with the hierarchical organization of the visual system, where early retinotopic cortex is more strongly constrained by local stimulus-driven temporal structure, whereas higher-order category-selective regions integrate more abstract and distributed representations [18,19].

While the entropy-based quantities provide a direct information-theoretic characterization of forecastability, the practical interpretation of conventional forecasting metrics requires additional context. The absolute RMSE values should be interpreted cautiously because all ROI time series were normalized prior to forecasting. Consequently, RMSE does not correspond directly to BOLD signal amplitudes or scanner acquisition accuracy. Because each ROI time series was standardized to have unit variance, an RMSE of 0.721 indicates that the predicted signal deviates from the true normalized signal by approximately 0.72 standard deviations on average. As commonly done in forecasting studies [13], RMSE is therefore used primarily as a relative measure for comparing forecasting models and quantifying improvement relative to baseline predictors. Relative to the naive persistence model, the transformer forecasting model reduced the hold-out test RMSE from 0.859 to 0.721, corresponding to an approximately 14% reduction in forecasting error. From an information-theoretic perspective, this improvement was accompanied by a predictive information fraction of η=75.49%, indicating that a substantial portion of future BOLD uncertainty could be explained from recent signal history.

Interestingly, forecasting performance was broadly comparable across both relatively simple and substantially more complex forecasting architectures. Linear regression, transformer, and LSTM models achieved similar validation behavior despite their differing representational capacities and modeling assumptions. From an information-theoretic perspective, the models also impose distinct assumptions regarding the underlying signal structure. Linear regression is naturally associated with approximately linear Gaussian dependencies; exponential smoothing emphasizes local temporal continuity, whereas neural architectures such as LSTMs and transformers can represent nonlinear and higher-dimensional temporal interactions. Nevertheless, all models remained constrained by the selected temporal memory window (M=50) and forecasting horizon (H=3), which define the effective temporal context available for prediction. The comparable forecasting performance across model classes therefore suggests that, within these experimental constraints, higher-capacity nonlinear architectures did not provide a substantial predictive advantage over simpler approaches. However, this finding should not be interpreted as evidence that the underlying BOLD dynamics are strictly linear or low-order. Nonlinear dependencies may still be present but remain difficult to detect using the present ROI-level representation, forecasting horizon, and evaluation metrics, which may not fully capture differences in the types of temporal structure exploited by different model classes.

The post hoc directed information analysis further clarified the structure of the learned predictive dependencies. The predominantly diagonal DI matrices indicate that the future activity of a given ROI is primarily associated with its own recent temporal history rather than with the histories of other ROIs. This finding is consistent with the autoregressive nature of the best-performing forecasting models and suggests that short-horizon BOLD prediction in this setting is dominated largely by local temporal persistence. Notably, ROIs 10 and 11, corresponding to bilateral V3B, exhibited the largest diagonal DI values, indicating particularly strong self-predictive information within this intermediate dorsal visual region. This may reflect relatively stable local temporal structure or sustained stimulus-driven activity in V3B, which is known to participate in higher-order retinotopic processing and integration of visual motion and spatial information [18,19]. Interestingly, despite the well-established hierarchical organization of the visual cortex, the DI analysis did not reveal a strong feedforward or hierarchical off-diagonal interaction structure under the selected temporal memory and forecasting settings. Instead, the dominant predictive structure remained largely local and autoregressive. Nevertheless, smaller but nonzero off-diagonal DI values remained observable across several ROI pairs, including interactions between VO2 and FFA, suggesting weaker distributed statistical dependencies between higher-order ventral visual regions. These interactions may reflect broader network-level integration processes that are not fully captured by purely local autoregressive dynamics. A sensitivity analysis using neighboring values of the forecasting memory length and prediction horizon produced qualitatively similar DI patterns, indicating that the predominantly diagonal structure was not an artifact of the specific parameter choice M = 50 and H = 3. While alternative temporal scales may reveal additional network-level interactions beyond those examined here, the present results suggest that short-timescale predictive structure in the selected visual ROIs is consistently dominated by local autoregressive dynamics.

More broadly, the present framework illustrates how information-theoretic quantities can provide interpretable summaries of learned neural forecasting dynamics. Even for comparatively high-capacity models such as transformers, entropy decomposition and directed information analyses yielded interpretable measures of uncertainty reduction, predictability, and directional dependence. In this sense, the forecasting models function not only as predictive tools, but also as data-driven probes for characterizing the statistical organization of large-scale fMRI activity.

### Limitations

This study has several limitations. Most notably, the forecasting horizon was limited to H=1,3,5 future samples, since longer horizons resulted in substantially increased validation loss and unstable predictive performance. Consequently, the present findings primarily characterize short-timescale temporal dependencies in BOLD activity. Future work could investigate multiscale or hierarchical forecasting architectures capable of maintaining stable probabilistic predictions over longer temporal horizons, while also incorporating richer network-level or latent-state representations.

The comparable performance of linear and nonlinear forecasting models should also be interpreted cautiously. Although higher-capacity models did not substantially improve forecasting performance in the present analysis, this does not rule out nonlinear dependencies in the underlying BOLD dynamics. Such dependencies may be obscured by ROI-level averaging, limited forecasting horizons, or evaluation metrics that emphasize aggregate prediction accuracy. Future work could incorporate metrics specifically sensitive to nonlinear dependence, nonlinear residual structure, or nonlinear predictive causality to better characterize model-class-specific contributions. At the same time, the primary goal of this work was to investigate the forecastability of fMRI signals and the extent of temporal redundancies present in the data. From this perspective, the forecasting models considered here were sufficient to characterize the predictive structure accessible under the studied conditions, even if additional nonlinear dependencies remain to be explored in future work.

An additional limitation arises from the histogram-based discretization procedure used for estimating predictive probability distributions and entropy-based quantities. The underlying BOLD forecasting distributions are continuous-valued, whereas entropy and directed information estimation in the present study were performed using discretized empirical histogram approximations primarily to simplify likelihood estimation and numerical computation. As a consequence, the absolute entropy values reported in nats depend on the selected discretization resolution, specifically the 100-bin histogram partition used throughout this work. In particular, finer histogram resolutions generally increase the estimated entropy values due to the increased partition cardinality. Accordingly, the reported entropy magnitudes should be interpreted relative to the discretization scheme employed rather than as absolute continuous entropy measures. Future work could instead employ continuous probabilistic density models and differential entropy estimators to obtain discretization-independent information-theoretic quantities.

## 5. Conclusions

In this work, we formulated multiregional fMRI BOLD forecasting as a probabilistic time-series prediction problem grounded in information theory. Using ROI-level activity from the Natural Scenes Dataset, we evaluated multiple forecasting architectures and quantified predictive structure through entropy-based measures and directed information analysis. The results demonstrated that short-horizon BOLD activity contains significant nontrivial temporal predictability beyond the naive persistence baseline, while still retaining substantial stochastic variability.

Entropy decomposition revealed that a considerable fraction of future uncertainty could be reduced using recent ROI history, and post hoc directed information analysis indicated that short-timescale prediction was dominated primarily by local autoregressive structure. Importantly, the study also demonstrated that information-theoretic quantities can provide interpretable summaries of learned dynamics even for comparatively complex black-box forecasting models.

Overall, the presented framework connects probabilistic forecasting, information theory, and multivariate neuroimaging analysis within a unified setting. These results suggest that forecasting-based approaches may provide a useful tool for studying the predictability, complexity, and directional organization of large-scale brain dynamics.

## Figures and Tables

**Table 1:** Regions of interest and their description.

Index (−/+)	ROI	MNI (x,y,z)	Radius
1	V1	(0,−90,0)	5
2/3	V2	(±10,−85,5)	5
4/5	V3	(±15,−80,10)	5
6/7	hV4	(±25,−75,−10)	6
8/9	V3A	(±20,−85,25)	6
10/11	V3B	(±25,−80,30)	6
12/13	LO1	(±35,−75,−5)	6
14/15	LO2	(±40,−75,−5)	6
16/17	VO1	(±25,−70,−15)	6
18/19	VO2	(±30,−65,−15)	6
20/21	PPA	(±28,−45,−12)	6
22/23	FFA	(±40,−55,−15)	6

**Table 2:** Forecasting performance across models for cross-validation and hold-out test evaluation.

Model	Cross-Validation		Hold-Out Test	
	RMSE	RMSSE	RMSE	RMSSE
Naive Last Value	0.920	1.39	0.859	1.47
Linear Regression	**0.767**	**1.17**	**0.710**	**1.22**
Exponential Smoothing	0.891	1.35	0.846	1.45
LSTM	0.779	1.18	0.733	1.26
Transformer	0.770	**1.17**	0.721	1.23

Best results within each evaluation setting are shown in bold.

## Data Availability

The Natural Scenes Dataset (NSD) analyzed in this study is publicly available from the original NSD release [17]. The code used for forecasting, entropy estimation, and directed information analysis is publicly available at https://github.com/ab126/fmri_forecasting (accessed on 21 June 2026). An archived release of the code associated with this manuscript is available through Zenodo: https://doi.org/10.5281/zenodo.20341604.

## Abbreviations

The following abbreviations are used in this manuscript:

BOLD	Blood-oxygen-level-dependent
CV	Cross-validation
DI	Directed Information
fMRI	Functional Magnetic Resonance Imaging
GenAI	Generative Artificial Intelligence
LOSO	Leave-one-subject-out
LSTM	Long Short-Term Memory
MNI	Montreal Neurological Institute
NSD	Natural Scenes Dataset
ROI	Region of Interest
